# Effect of Vitamin D_3_ Supplementation on Acute Fracture Healing: A Phase II Screening Randomized Double‐Blind Controlled Trial

**DOI:** 10.1002/jbm4.10705

**Published:** 2022-12-22

**Authors:** Gerard P. Slobogean, Sofia Bzovsky, Nathan N. O'Hara, Lucas S. Marchand, Zachary D. Hannan, Haley K. Demyanovich, Daniel W. Connelly, Jonathan D. Adachi, Lehana Thabane, Sheila Sprague, the Vita‐Shock Investigators

**Affiliations:** ^1^ R Adams Cowley Shock Trauma Center, Department of Orthopaedics University of Maryland School of Medicine Baltimore MD USA; ^2^ Division of Orthopaedic Surgery, Department of Surgery McMaster University Hamilton Canada; ^3^ University Orthopaedic Center University of Utah Salt Lake City UT USA; ^4^ Department of Medicine McMaster University Hamilton Canada; ^5^ Department of Health Research Methods, Evidence, and Impact McMaster University Hamilton Canada

**Keywords:** CLINICAL TRIALS, EPIDEMIOLOGY, INJURY/FRACTURE HEALING, NUTRITION, ORTHOPEDICS

## Abstract

Nearly half of adult fracture patients are vitamin D deficient (serum 25‐hydroxyvitamin D [25(OH)D] levels <20 ng/mL). Many surgeons advocate prescribing vitamin D supplements to improve fracture healing outcomes; however, data supporting the effectiveness of vitamin D_3_ supplements to improve acute fracture healing are lacking. We tested the effectiveness of vitamin D_3_ supplementation for improving tibia and femur fracture healing. We conducted a single‐center, double*‐*blinded phase II screening randomized controlled trial with a 12‐month follow‐up. Patients aged 18–50 years receiving an intramedullary nail for a tibia or femoral shaft fracture were randomized 1:1:1:1 to receive (i) 150,000 IU loading dose vitamin D_3_ at injury and 6 weeks (*n* = 27); (ii) 4000 IU vitamin D_3_ daily (*n* = 24); (iii) 600 IU vitamin D_3_ daily (*n* = 24); or (iv) placebo (*n* = 27). Primary outcomes were clinical fracture healing (Function IndeX for Trauma [FIX‐IT]) and radiographic fracture healing (Radiographic Union Score for Tibial fractures [RUST]) at 3 months. One hundred two patients with a mean age of 29 years (standard deviation 8) were randomized. The majority were male (69%), and 56% were vitamin D_3_ deficient at baseline*.* Ninety‐nine patients completed the 3‐month follow‐up. In our prespecified comparisons, no clinically important or statistically significant differences were detected in RUST or FIX‐IT scores between groups when measured at 3 months and over 12 months. However, in a post hoc comparison, high doses of vitamin D_3_ were associated with improved clinical fracture healing relative to placebo at 3 months (mean difference [MD] 0.90, 80% confidence interval [CI], 0.08 to 1.79; *p* = 0.16) and within 12 months (MD 0.89, 80% CI, 0.05 to 1.74; *p* = 0.18). The study was designed to identify potential evidence to support the effectiveness of vitamin D_3_ supplementation in improving acute fracture healing. Vitamin D_3_ supplementation, particularly high doses, might modestly improve acute tibia or femoral shaft fracture healing in healthy adults, but confirmatory studies are required. The Vita‐Shock trial was awarded the Orthopaedic Trauma Association's (OTA) Bovill Award in 2020. This award is presented annually to the authors of the most outstanding OTA Annual Meeting scientific paper. © 2022 The Authors. *JBMR Plus* published by Wiley Periodicals LLC on behalf of American Society for Bone and Mineral Research.

## Introduction

Tibial and femoral shaft fractures are routinely treated with intramedullary nail fixation. Complications following these injuries include delayed healing and nonunion, which may require additional surgical procedures.^(^ ^1^, ^2^, ^3^ ^)^ Ultimately, these fracture healing complications lead to diminished function and quality of life.^(^ ^4^, ^5^, ^6^, ^7^ ^)^

Research suggests that circulating vitamin D metabolite concentrations may affect fracture healing, with inadequate vitamin D causing altered bone metabolism and slower fracture healing.^(^ ^8^, ^9^, ^10^, ^11^, ^12^ ^)^ Nearly half of adult fracture patients are vitamin D deficient with serum 25‐hydroxyvitamin D [25(OH)D] levels <20 ng/mL.^(^ ^13^, ^14^ ^)^ In response, many surgeons advocate prescribing vitamin D_3_ supplements to improve fracture healing outcomes, even in healthy nonosteoporotic patients.^(^ ^15^ ^)^ Although healthcare guidelines suggest that adults should take vitamin D_3_ supplements for general bone health and osteoporosis prevention,^(^ ^16^ ^)^ there is sparse evidence to guide its use to improve fracture healing.

To address this knowledge gap, we conducted a phase II screening randomized controlled trial (RCT) to test the preliminary effectiveness of three potential vitamin D_3_ supplementation strategies for improving acute tibia and femur fracture healing in adults aged 18–50 years. We also report the association between 25(OH)D serum levels and fracture healing at 3 months, the adherence to the nutritional supplementation, and a post hoc comparison of the effectiveness of any vitamin D_3_ supplementation and high dose vitamin D_3_ supplementation compared to placebo.

## Materials and Methods

### Study design

We conducted a four‐arm, double‐blinded, phase II screening, randomized, placebo‐controlled trial comparing three vitamin D_3_ supplementation strategies in young adult patients with a tibial or femoral shaft fracture. Phase II screening trials provide preliminary evidence of a treatment benefit within a feasible sample size of an initial pilot study. Details of the trial protocol were published previously.^(^ ^17^ ^)^ The trial was registered with ClinicalTrials.gov, No. NCT02786498, and approved by the Hamilton Integrated Research Ethics Board (No. 2017‐1952) and the University of Maryland Institutional Review Board (No. HP‐00069705).

### Participants

We screened fracture patients treated at the R Adams Cowley Shock Trauma Center in Baltimore, MD. Eligible patients were adult men and women aged 18 to 50 years with an acute closed or low‐grade open (Gustilo type I or II) tibial or femoral shaft fracture managed with a reamed, locked intramedullary nail. This population was selected to create a relatively homogeneous population of lower‐extremity fractures expected to heal via callus formation and secondary bone healing. We excluded patients with osteoporosis, stress fractures, elevated serum calcium levels, atypical femur fractures,^(^ ^18^ ^)^ hyperhomocysteinemia, allergy or contraindication to being prescribed vitamin D_3_, or a known or likely undiagnosed bone metabolism disorder.^(^ ^17^ ^)^ All participants provided written informed consent.

### Treatment groups

Each participant was randomized 1:1:1:1 using a computer‐generated system to one of four treatment groups: (i) 150,000 IU loading dose vitamin D_3_ at injury and 6 weeks plus placebo daily, (ii) placebo loading doses plus 4000 IU vitamin D_3_ daily, (iii) placebo loading doses plus 600 IU vitamin D_3_ daily, or (iv) placebo loading and placebo daily doses. Participants were given a bottle of either active vitamin D_3_ or placebo capsules and were instructed to take one capsule daily for 3 months, commencing within 1 week of injury. The loading dose of vitamin D_3_ supplements or placebo were given within 1 week of injury and at 6 weeks (± 2 weeks) after injury in the hospital or the fracture clinic. The placebo capsules had no active ingredients and were visually identical to the vitamin D capsules. Bio‐Tech Pharmacal, Inc. (Fayetteville, AR) produced all vitamin D and placebo doses. Complete blinding of the treatment allocation to all participants and research staff was maintained throughout the entire trial.

### Fracture healing

Clinical and radiographic fracture healing at 3 months after randomization were selected as co‐primary outcomes. We selected these outcome measures and the 3‐month timepoint as primary outcomes for the greatest potential to detect clinical differences in early fracture healing and to coincide with the standard clinical follow‐up schedule.^(^ ^17^ ^)^ Clinical fracture healing was measured using the Function IndeX for Trauma (FIX‐IT), a standardized measure of weight bearing and pain in patients with lower‐extremity fractures.^(^ ^19^ ^)^ Radiographic fracture healing was measured using the Radiographic Union Score for Tibial fractures (RUST), which assesses the presence of bridging callus or a persistent fracture line on each of the four cortices.^(^ ^20^, ^21^, ^22^, ^23^ ^)^ Participants had an X‐ray taken at fracture clinic visits at 6 weeks, 3 months, 6 months, 9 months, and 12 months after fracture. An orthopedic surgeon, independent of the study and blinded to the treatment allocation, reviewed all available X‐ray images and assigned a RUST score. As a secondary outcome, we longitudinally assessed repeated measures of clinical and radiographic fracture healing over 12 months.

At the time of protocol development, we had also sought to assess biological fracture healing by measuring serum levels of cross‐linked C‐terminal telopeptides of type I collagen (CTX) and amino‐terminal procollagen propeptides of collagen type I (PINP).^(^ ^24^ ^)^ These two bone‐turnover markers were defined a priori as tertiary and quaternary measures of healing in our hierarchical definition of fracture healing; however, because reliable definitions of healing for these markers were never established, they were excluded from our primary analyses.

### 25(OH)D serum levels

Participants had blood drawn at enrollment and at 6 weeks and 3 months after fracture. These data were used to determine whether there was an association between fracture healing and a participant's 25(OH)D levels at enrollment, changes in 25(OH)D levels from randomization to 3 months, or 25(OH)D levels at 3 months.

### Adherence to supplementation

At hospital discharge, research personnel documented whether the participant received their daily supplement bottle and first supplement loading dose within 1 week of injury. At the 6‐week follow‐up visit, research personnel documented whether participants received their second loading dose at 6 weeks after injury. At the 6‐week and 3‐month follow‐up visits, participants were asked if they had missed any doses since their last follow‐up visit and if they were taking any additional vitamin D_3_ supplements other than the trial intervention.

## Statistical Analysis

### Sample size

The planned sample size for this trial was 96 patients (24 patients per treatment group). With an a priori increased acceptable type I error threshold of 20% (alpha), this sample size would provide 80% power to detect a two‐point difference in the FIX‐IT and RUST scores, assuming a three‐point standard deviation, for each hypothesized comparison: (i) high doses (loading or daily) will increase healing compared to low daily dose, (ii) high loading dose will increase healing compared to high daily dose, (iii) low daily dose will increase healing compared to placebo. Details of the sample size justification were published previously.^(^ ^17^ ^)^ We added two post hoc hypotheses, testing the healing outcomes between (i) any vitamin D_3_ supplementation versus placebo and (ii) high doses (loading or daily) versus placebo.

### Statistical analysis of outcomes

All analyses were exploratory and performed using the intention‐to‐treat principle and presented according to the statistical planning methodology proposed by Chan et al.^(^ ^25^ ^)^ The results are reported as an estimate of effect expressed as mean difference (MD), corresponding 80% confidence interval (CI), and associated *p* value. Given the exploratory nature of the study, we did not adjust for multiple testing. Missing data were imputed using multiple imputations. R (version 4.0.2) was used to perform all analyses.

### Fracture healing

Comparisons for our three fracture healing hypotheses at 3 months were made using independent *t*‐tests with the significance set at α = 0.20. The first hypothesis compared high dose supplementation versus low dose. To test this hypothesis, we combined the two high dose groups (150,000 IU loading and 4000 IU daily) for a 2:1 comparison against the low (600 IU) daily dose group. The other two a priori comparisons were high loading dose versus high daily dose and low dose versus placebo. Each measure of fracture healing was described with its mean and standard deviation (SD). Using multilevel repeated‐measure models, the effect of the vitamin D_3_ dose on fracture healing outcomes (FIX‐IT and RUST) over 12 months were also estimated, with randomized treatment, visit, and fracture location included as independent variables. Additional subgroup analyses were conducted to determine whether differential treatment effects existed within the vitamin D_3_‐deficient population. Finally, post hoc comparisons of fracture healing at 3 months and over the 12‐month study period between patients that received any dose of vitamin D_3_ or high dose vitamin D_3_ supplementation versus placebo were performed.

### 25(OH)D serum levels

To determine whether 25(OH)D serum levels were associated with fracture healing at 3 months, we conducted a linear regression analysis, where 25(OH)D serum level was the dependent variable and fracture healing was the independent variable, with significance set at α = 0.20. Specifically, we evaluated whether associations existed between fracture healing and (i) participants' enrollment serum 25(OH)D level, (ii) their change in 25(OH)D from enrollment to 3 months, and (iii) their 25(OH)D level at 3 months. 25(OH)D serum levels at baseline and at 6 weeks and 3 months after fracture were presented for each treatment group as means and SDs.

## Results

### Enrollment

**Fig. 1:**
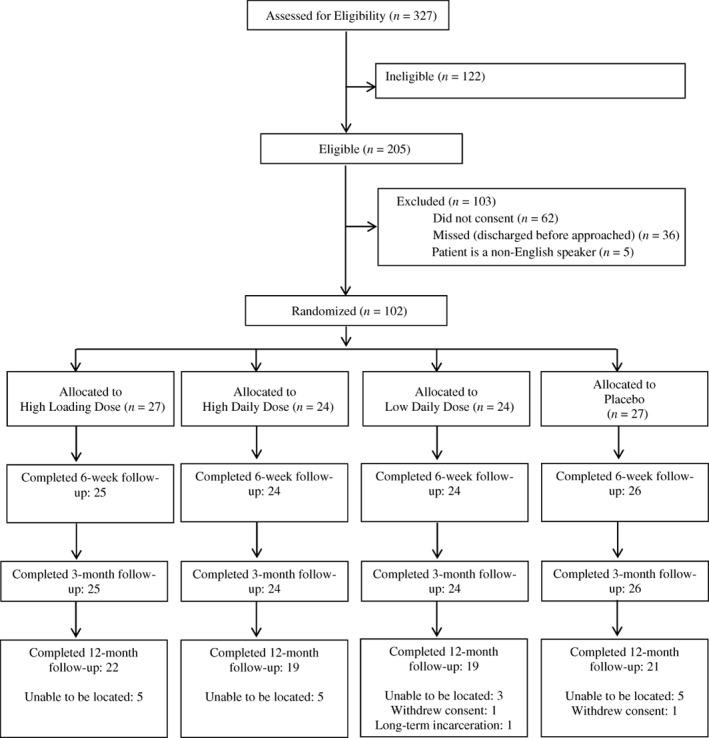
Participant flow diagram.

Between November 2016 and May 2019, 327 patients were screened for participation, and 102 patients were eligible and included (Fig. 1 and Table 1). Of the 102 participants, 27 were allocated to the high loading (150,000 IU) dose group, 24 to the high daily (4000 IU) dose group, 24 to the low daily (600 IU) dose group, and 27 to the placebo group. Ninety‐nine participants (97.1%) completed the follow‐up at 3 months, and 81 (79.4%) completed the 12‐month follow‐up.

**Table 1:** Reasons for Exclusion^a^

Reason for exclusion	Statistics count (%) *n* = 225
Patient did not consent	62 (27.7)
Other lower‐extremity injuries that prevented full weight bearing by 6 weeks after fracture	53 (23.7)
Patients who would likely have had problems with maintaining follow‐up	39 (17.4)
Missed (patient was admitted and discharged over the weekend when the research pharmacy was unavailable)	36 (16.1)
Incarcerated patients	8 (3.6)
Patients taking over‐the‐counter multivitamins that contain vitamin D and were unable or unwilling to discontinue their use for the study	8 (3.6)
Patient is a non‐English speaker	5 (2.2)
Patients who were not expected to survive their injuries	4 (1.8)
Patient not enrolled within 7 days of injury	2 (0.9)
Patients with known or likely undiagnosed disorders of bone metabolism	2 (0.9)
Pregnancy	2 (0.9)
Pathological fractures secondary to neoplasm or other bone lesion	1 (0.4)
Patient under the age of 18 or over the age of 50	1 (0.4)
Patients with an allergy to vitamin D or another contraindication to being prescribed vitamin D	1 (0.4)
Gustilo type III open fracture	1 (0.4)

^a^ One reason per patient based on order of reason for exclusion in table.

### Participant demographics and fracture characteristics

Most participants were male (69%), with a mean age of 29 years (SD 8). Sixty percent of the participants had femoral shaft fractures, and 40% had tibial shaft fractures. Just over half (56%) of participants were vitamin D_3_ deficient at baseline, with no qualitative differences observed between the groups (Table 2).

**Table 2:** Participant Demographics and Fracture Characteristics

Characteristic	150,000 IU loading + placebo daily *n* = 27	Placebo loading +4000 IU daily *n* = 24	Placebo loading +600 IU daily *n* = 24	Placebo loading + placebo daily *n* = 27
Age, mean (SD) years	27.4 (8.1)	28.8 (7.3)	31.1 (9.8)	31.3 (7.9)
Gender, *n* (%)				
Male	14 (51.9)	19 (79.2)	16 (66.7)	21 (77.8)
BMI, (kg/m^2^), *n* (%)				
Underweight <18.5	0 (0.0)	0 (0.0)	0 (0.0)	1 (3.7)
Normal weight 18.5–24.9	8 (29.6)	10 (41.7)	13 (54.2)	15 (55.6)
Overweight 25–29.9	7 (25.9)	7 (29.2)	6 (25.0)	10 (37.0)
Obese 30–39.9	9 (33.3)	7 (29.2)	4 (16.7)	1 (3.7)
Morbidly obese ≥40	3 (0.0)	0 (0.0)	1 (4.2)	0 (0.0)
Ethnicity, *n* (%)				
White	14 (51.9)	11 (45.8)	13 (54.2)	10 (37.0)
Black	11 (40.7)	11 (45.8)	10 (41.7)	15 (55.6)
Hispanic/Latino	2 (7.4)	2 (8.3)	0 (0.0)	1 (3.7)
South Asian	0 (0.0)	0 (0.0)	0 (0.0)	1 (3.7)
East Asian	0 (0.0)	0 (0.0)	1 (4.2)	0 (0.0)
Smoking status, *n* (%)				
Current smoker	10 (37.0)	6 (25.0)	7 (29.2)	14 (51.9)
Previous smoker	1 (3.7)	3 (12.5)	2 (8.3)	0 (0.0)
Nonsmoker	16 (59.3)	15 (62.5)	15 (62.5)	13 (48.1)
Fractured bone, *n* (%)				
Tibia	11 (40.7)	9 (37.5)	10 (41.7)	11 (40.7)
AO classification, *n* (%)				
32.A	10 (37.0)	6 (25.0)	9 (37.5)	9 (33.3)
32.B	4 (14.8)	4 (16.7)	5 (20.8)	3 (11.1)
32.C	2 (7.4)	4 (16.7)	2 (8.3)	4 (14.8)
42.A	7 (25.9)	3 (12.5)	3 (12.5)	6 (22.2)
42.B	3 (11.1)	3 (12.5)	4 (16.7)	3 (11.1)
42.C	1 (3.7)	4 (16.7)	1 (4.2)	2 (7.4)
Degree of soft tissue injury, *n* (%)				
Tscherne classification	*N* = 20	*N* = 17	*N* = 18	*N* = 20
0	6 (30.0)	3 (17.6)	3 (16.7)	6 (30.0)
1	11 (55.0)	12 (70.6)	11 (61.1)	8 (40.0)
2	3 (15.0)	1 (5.9)	3 (16.7)	5 (25.0)
3	0 (0.0)	1 (5.9)	1 (5.6)	1 (5.0)
Gustilo classification	N = 7	N = 7	N = 6	N = 7
I	3 (42.9)	2 (28.6)	5 (83.3)	4 (57.1)
II	4 (57.1)	5 (71.4)	1 (16.7)	3 (42.9)
Vitamin D_3_ deficient (serum 25(OH)D serum levels <20 ng/mL), *n* (%)	16 (59.3)	11 (45.8)	12 (50.0)	17 (62.3)
Baseline serum 25(OH)D levels, mean (SD) (ng/mL)	18.8 (7.8)	20.7 (9.6)	19.7 (7.9)	17.3 (7.5)

25(OH)D = 25‐hydroxyvitamin D; BMI = body mass index; IU = international units; SD = standard deviation.

### Fracture healing at 3 months

At 3 months, the mean FIX‐IT score was 9.5 out of 12 (SD 2.8). The differences in our three a priori hypotheses did not reach our threshold for statistical significance, including comparisons between the high dose groups versus low dose groups (MD 0.60, 80% CI −0.34, 1.65; *p* = 0.40), high loading dose versus high daily dose (MD 0.40, 80% CI −1.32, 0.60; *p* = 0.62), and low dose versus placebo (MD 0.30, 80% CI −1.41, 0.84; *p* = 0.74) (Table 3). However, our post hoc comparison of high dose versus placebo was statistically significant at our prespecified threshold (MD 0.90, 80% CI, 0.08 to 1.79; *p* = 0.16).

**Table 3:** Fracture Healing Outcomes at 3 Months

Hypothesis 1	High dose, *n* = 51	Low dose, *n* = 24	Mean difference (80% CI)	*p* value
FIX‐IT score, mean (SD)	9.9 (2.6)	9.3 (3.3)	0.60 (−0.34, 1.65)	0.40
RUST score, mean (SD)	11.1 (2.8)	11.0 (3.0)	0.10 (−0.77, 1.13)	0.80

Hypothesis 2	High loading dose, *n* = 27	High daily dose, *n* = 24	Mean difference (80% CI)	*p* value
FIX‐IT score, mean (SD)	10.1 (2.5)	9.7 (2.8)	0.40 (−0.60, 1.32)	0.62
RUST score, mean (SD)	11.0 (2.8)	11.3 (2.9)	−0.30 (−1.25, 0.83)	0.79

Hypothesis 3	Low dose, *n* = 24	Placebo, *n* = 27	Mean difference (80% CI)	*p* value
FIX‐IT score, mean (SD)	9.3 (3.3)	9.0 (2.9)	0.30 (−0.84, 1.41)	0.74
RUST score, mean (SD)	11.0 (3.0)	10.4 (2.5)	0.60 (−0.49, 1.52)	0.51

Post hoc analysis 1	Any vitamin D dose, *n* = 75	Placebo, *n* = 27	Mean difference (80% CI)	*p* value
FIX‐IT score, mean (SD)	9.7 (2.8)	9.0 (2.9)	0.70 (−0.09, 1.55)	0.25
RUST score, mean (SD)	11.1 (2.9)	10.4 (2.5)	0.70 (−0.16, 1.44)	0.31

Post hoc analysis 2	High dose, *n* = 51	Placebo, *n* = 27	Mean difference (80% CI)	*p* value
FIX‐IT score, mean (SD)	9.9 (2.6)	9.0 (2.9)	0.90 (0.08, 1.79)	0.16
RUST score, mean (SD)	11.1 (2.8)	10.4 (2.5)	0.70 (−0.79, 1.28)	0.76

CI = confidence interval; FIX‐IT = function index for trauma; RUST = radiographic union score for tibial fractures; SD = standard deviation.

The mean RUST score at 3 months was 10.9 out of 12 (SD 2.8). We did not observe significant differences in our prespecified comparisons between the high dose groups versus low dose group (MD 0.10, 80% CI −0.77, 1.13; *p* = 0.80), high loading dose versus high daily dose (MD −0.30, 80% CI −0.83, 1.25; *p* = 0.79), and low dose versus placebo (MD 0.60, 80% CI −1.52, 0.49; *p* = 0.51). Similar results were observed for the post hoc comparison of all vitamin D_3_ supplementation doses and high dose supplementation versus placebo (Table 3).

The largest between‐group mean difference at 3 months in RUST scores was 0.90 (80% CI −0.18 to 1.79) when comparing the high daily dose group to the placebo group and 1.10 (80% CI 0.17 to 2.05) for FIX‐IT scores when comparing the high loading dose group to the placebo group. Additional subgroup analyses within vitamin D deficient patients also showed no difference in RUST or FIX‐IT scores between groups.

### Fracture healing over 12 months

Over 12 months, no clinically important or statistically significant differences were detected in RUST or FIX‐IT scores for the prespecified comparisons (Table 4). However, we did observe a modest increase in the FIX‐IT score with high dose supplementation compared to placebo (MD 0.89, 80% CI, 0.05 to 1.74; *p* = 0.18).

**Table 4:** Fracture Healing Outcomes over 12 Months

	Adjusted mean difference^a^ (80% CI)	*p* value
Hypothesis 1—High dose versus low dose		
FIX‐IT score	0.59 (−0.17, 1.36)	0.32
RUST score	0.51 (−0.22, 1.24)	0.37

Hypothesis 2—High loading dose versus high daily dose		
FIX‐IT score	0.44 (−0.44, 1.31)	0.52
RUST score	−0.19 (−1.01, 0.62)	0.76

Hypothesis 3—Low dose versus placebo dose		
FIX‐IT score	0.28 (−0.79, 0.93)	0.73
RUST score	−0.55 (−1.40, 0.29)	0.40

Post hoc analysis 1—Any vitamin D dose versus placebo dose		
FIX‐IT score	0.70 (−0.08, 1.48)	0.25
RUST score	−0.21 (−0.86, 0.44)	0.68

Post hoc analysis 2—High dose versus placebo dose		
FIX‐IT score	0.89 (0.05, 1.74)	0.18
RUST score	−0.04 (−0.73, 0.63)	0.93

CI = confidence interval; FIX‐IT = function index for trauma; RUST = radiographic union score for tibial fractures; SD = standard deviation.

^a^ Analysis adjusted for fracture location (tibia versus femur).

### 25(OH)D serum levels

**Fig. 2:**
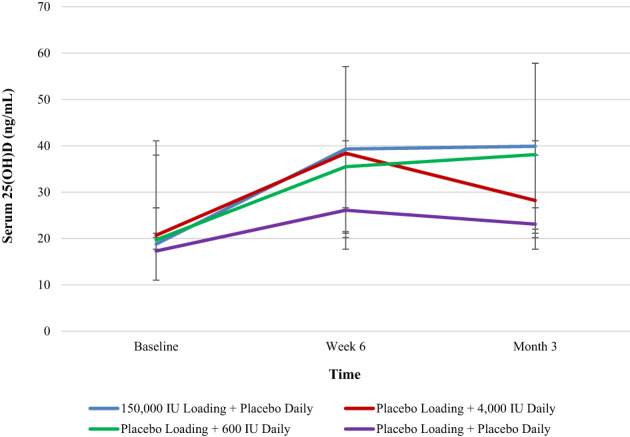
Serum 25(OH)D concentration by treatment group.

25(OH)D serum levels increased from enrollment to the 6‐week visit in all groups (Fig. 2). The changes in 25(OH)D serum levels from baseline to 6 weeks were significantly higher in the three vitamin D_3_ treatment groups relative to the placebo group (high loading versus placebo, MD 11.9 ng/mL, 80% CI, 5.8 to 18.0; *p* = 0.01; high daily versus placebo, MD 9.4 ng/mL, 80% CI, 3.0 to 15.7; *p* = 0.06; low daily versus placebo, MD 7.3 ng/mL, 80% CI, 1.0 to 13.6; *p* = 0.14). There was little change in the 25(OH)D serum levels from the 6‐week to 3‐month visits. Despite vitamin D_3_ supplementation increasing serum 25(OH)D concentrations, there was no evidence to support the relationship between serum levels and fracture healing (*p* > 0.20) (Table 5).

**Table 5:** Associations Between Fracture Healing and 25(OH)D Serum Concentration

Outcome measure	Estimated beta coefficient (80% CI)	*p* value
FIX‐IT Score at 3 months		
Enrollment serum 25(OH)D concentration	0.002 (−0.04, 0.05)	0.95
Change in serum 25(OH)D concentration from baseline to 3 months	0.009 (−0.01, 0.03)	0.51
Serum 25(OH)D concentration at 3 months	0.01 (−0.01, 0.03)	0.48
RUST score		
Enrollment serum 25(OH)D concentration	−0.03 (−0.07, 0.02)	0.44
Change in serum 25(OH)D concentration from baseline to 3 months	0.01 (−0.01, 0.03)	0.31
Serum 25(OH)D concentration at 3 months	0.01 (−0.01 0.03)	0.48

25(OH)D = 25‐hydroxyvitamin D; CI = confidence interval; FIX‐IT = function index for trauma; RUST = radiographic union score for tibial fractures.

### Supplementation adherence

Ninety‐eight percent of participants received their first loading dose within 1 week of injury, and 84% received their second loading dose at 6 weeks after injury. In addition, 78% of participants reported taking a minimum of 75% of their prescribed daily supplements at both the 6‐week and 3‐month follow‐up periods. Eighteen percent of participants self‐reported full adherence to their daily supplementation for the entire 3‐month period (Table 6).

**Table 6:** Supplementation Adherence

Measure/Timing	150,000 IU loading + placebo daily *n* = 27	Placebo loading +4000 IU daily *n* = 24	Placebo loading +600 IU daily *n* = 24	Placebo loading + placebo daily *n* = 27
Provided with daily supplement bottle, *n* (%)	27 (100.0)	24 (100.0)	24 (100.0)	26 (96.3)
Received daily supplement bottle within 1 week of injury, *n* (%)	26 (96.3)	24 (100.0)	24 (100.0)	26 (96.3)
Received first supplement loading dose within 1 week of injury, *n* (%)	26 (96.3)	24 (100.0)	24 (100.0)	26 (96.3)
Adherence to daily supplementation, *n* (%)^a^				
6 weeks	19 (70.4)	20 (83.3)	18 (75.0)	22 (81.5)
3 months	21 (77.8)	19 (79.2)	19 (79.2)	20 (74.1)
Fully compliant with 3‐month dosing, *n* (%)	4 (14.8)	5 (20.8)	5 (20.8)	4 (14.8)
Number of missed doses based on self‐report, *n* (%)				
6 weeks				
≤10 missed	5 (18.5)	8 (33.3)	8 (33.3)	8 (29.6)
11–25 missed	2 (7.4)	0 (0.0)	4 (16.7)	2 (7.4)
26–50 missed	4 (14.8)	5 (20.8)	5 (20.8)	2 (7.4)
51–75 missed	2 (7.4)	0 (0.0)	0 (0.0)	0 (0.0)
3 months				
≤10 missed	9 (33.3)	6 (25.0)	6 (25.0)	9 (33.3)
11–25 missed	2 (7.4)	1 (41.7)	2 (8.3)	3 (11.1)
26–50 missed	2 (7.4)	4 (16.7)	1 (4.2)	1 (4.2)
51–75 missed	0 (0.0)	2 (8.3)	1 (4.2)	0 (0.0)
76–100 missed	0 (0.0)	1 (4.2)	1 (4.2)	4 (16.7)
Received second supplement loading dose at 6 weeks after injury, *n* (%)	23 (85.2)	19 (79.2)	22 (91.7)	22 (81.5)
Use of additional vitamin D_3_ supplements other than trial intervention, *n* (%)				
6 weeks				
Daily dose	0 (0.0)	4 (16.7)	4 (16.7)	1 (4.2)
Loading dose	0 (0.0)	0 (0.0)	0 (0.0)	0 (0.0)
3 months				
Daily dose	0 (0.0)	3 (12.5)	0 (0.0)	2 (7.4)
Loading dose	0 (0.0)	0 (0.0)	0 (0.0)	0 (0.0)

^a^ Adherence to daily vitamin D_3_ supplementation is defined as those who took at least 75% of their daily supplementation.

## Discussion

This study was designed to identify potential evidence to support the effectiveness of vitamin D_3_ supplementation to improve acute fracture healing and guide the design of a future definitive comparative trial. Based on the results of this phase II screening trial and other available evidence, it remains unclear whether vitamin D_3_ supplementation improves fracture healing in nonosteoporotic patients.^(^ ^26^, ^27^, ^28^, ^29^, ^30^, ^31^, ^32^, ^33^, ^34^, ^35^ ^)^ The use of vitamin D_3_ supplementation did improve serum 25(OH)D levels, especially from enrollment to 6 weeks after fracture in all supplementation groups, and our post hoc comparisons suggest high dose supplementation might modestly improve early clinical fracture healing.

The results of our trial contribute to a growing series of RCTs investigating the potential fracture healing benefits of vitamin D_3_ supplementation. In a recent three‐arm RCT, Heyer et al. randomized 32 postmenopausal women with a nonoperatively managed distal radius fracture to receive oral vitamin D_3_ boluses at 1–2 weeks and 6–8 weeks after injury.^(^ ^36^ ^)^ Eleven participants received 75,000 IU boluses, 10 participants received 30,000 IU boluses, and 11 participants received no supplementation (control group). The authors compared the treatment outcomes using high‐resolution peripheral quantitative computed tomography (HR‐pQCT) and microfinite element analysis‐derived torsion, compression, and bending stiffness. In this population of women without vitamin D deficiency, both intervention groups achieved higher serum 25(OH)D levels than the control group; however, no fracture healing benefits of vitamin D_3_ supplementation were detected. Instead, a possibly detrimental effect of decreased trabecular number, lower compression stiffness, and increased bone resorption was identified with the high dose bolus treatment.

The results of our trial extend many of the findings of Heyer et al. The differences in trabeculae, compression stiffness, and bone resorption seen in their distal radius population are relevant for metaphyseal bone healing; however, no differences were detected between their groups for comparisons of bone formation, bending stiffness, or torsion—all properties typically correlated with fracture callus and cortical diameter. Since our study examines tibial and femoral shaft fracture healing, it could be expected that cortical bone formation and mechanical stiffness would be more important in our diaphyseal healing outcomes. This result is consistent with our study's findings suggesting no differences in radiographic healing between the treatment groups.

Two other RCTs failed to demonstrate any clinical fracture healing benefits from vitamin D_3_ supplementation. Haines et al. randomized 100 long bone fracture patients to receive 100,000 IU vitamin D_3_ or placebo and found no difference in nonunion between treatment groups (4% versus 4%, *p* = 1.00).^(^ ^35^ ^)^ Similarly, our group completed the FAITH‐2 pilot trial, which compared 4000 IU vitamin D_3_ versus placebo in adult hip fracture patients younger than 60 years. Among patients with ipsilateral femoral shaft fractures, the FAITH‐2 results detected no differences in the average time to radiographic femoral shaft healing between the groups (*p* = 0.52).^(^ ^26^ ^)^ Furthermore, vitamin D supplementation did not reduce the risk of other patient‐important complications associated with fracture healing.^(^ ^26^ ^)^

Despite sparse data to suggest vitamin D_3_ supplementation improves acute fracture healing in nonosteoporotic fractures, many clinicians believe vitamin D supplements should be routinely prescribed to all healthy adult fracture patients.^(^ ^30^ ^)^ Given the low cost of vitamin D supplements and their robust safety profile, this approach would be a simple practice to implement. This policy would also overcome current controversies regarding various serum 25(OH)D thresholds used to initiate supplementation. This is particularly important given data from an observational cohort study of tibial and femoral shaft fractures noted normal physiologic drops in serum 25(OH)D concentrations between initial emergency department measurement and repeat measurements 1 week later.^(^ ^37^ ^)^ These findings suggest the lower observed 25(OH)D levels in fracture patients may be part of a normal initial physiologic response during early fracture healing. It may also explain why the placebo group also experienced a small increase in 25(OH)D, which may reflect their serum levels returning to their normal baseline. Regardless, the impetus for our trial was sparked by prior work demonstrating wide variability in the dose and duration of vitamin D used by orthopedic surgeons and a need to identify optimal supplementation strategies.^(^ ^15^ ^)^

This phase II screening trial provides essential data to address prior knowledge gaps in the use of vitamin D supplementation. Our current study compared multiple vitamin D_3_ dosing strategies that could be easily implemented; the current trial not only tested a loading dose strategy (150,000 IU), it also compared low (600 IU) and high (4000 IU) daily doses of vitamin D_3_. Moreover, we considered both radiographic and clinical healing and used a 3‐month endpoint to look for early signals of more rapid healing progression and a 12‐month time course for sustained differences. Finally, we expanded our prior investigation of vitamin D_3_ from adult femoral neck fracture patients to long bone fractures of the femur and tibia.^(^ ^31^ ^)^

In addition to our four‐arm approach to test several vitamin D_3_ supplementation dosing strategies, several other aspects of our study design strengthened our study conclusions. First, we minimized bias by using double‐blinded randomization and a placebo control. Second, implementing a phase II screening study design with a larger alpha threshold allowed us to screen for any potential vitamin D_3_ effect to guide future definitive RCTs. Despite these benefits of our study design, the relatively small sample size can still lead to type II error. Similarly, the 3‐month timepoint for the primary analysis may be too delayed for detecting early fracture healing benefits, and perhaps a 6‐week assessment might have been more sensitive. Additionally, although the double‐blind nature of the study protects against many potential assessment biases, it does not protect against inadequate adherence to the prescribed vitamin D_3_ supplementation strategies. In our study, 78% of participants reported taking a minimum of 75% of their prescribed daily supplements at both the 6‐week and 3‐month follow‐up periods. Finally, we further explored our data to look for potential differential treatment effects based on patients' baseline vitamin D deficiency and a comparison of any vitamin D_3_ and high doses of supplementation versus placebo. These post hoc analyses suggest high doses of supplementation might provide a modest clinical fracture healing benefit to supplementation and further support our main findings. Further research is required to confirm this finding.

Overall, our results appear largely consistent with a mounting body of vitamin D research questioning previously hypothesized benefits of vitamin D supplementation for nonskeletal pathology.^(^ ^26^, ^31^, ^32^, ^33^, ^34^, ^35^ ^)^ However, our post hoc analyses suggest high doses of vitamin D_3_ might confer a modest benefit in clinical fracture healing, but this result requires confirmation in a larger RCT.

## Conflicts of Interest

Dr. Slobogean sits on the editorial or governing board of the *Journal of Orthopedic Trauma*, serves as a paid consultant for Nuvasive, is a board or committee member for the Orthopedic Trauma Association, receives research support from the Patient Centered Outcomes Research Institute, serves as a paid consultant for Smith & Nephew, receives research support from the US Department of Defense, and serves as a paid consultant for Zimmer, outside the submitted work. Dr. O'Hara reports stock or stock options from Arbutus Medical Inc., outside the submitted work. Dr. Adachi reports personal fees and research support from Amgen Co., serves as a board or committee member for the International Osteoporosis Foundation, sits on the editorial or governing board for Osteoporosis International, and receives research support from Radius, outside the submitted work. Dr. Sprague serves as a board or committee member for the Orthopedic Trauma Association, is employed by Global Research Solutions Inc., and receives consultant fees from the University of Sherbrooke and Platform Life Sciences, all outside the submitted work. All other coauthors have nothing to disclose.

### Peer Review

The peer review history for this article is available at https://publons.com/publon/10.1002/jbm4.10705.

## Data Availability

Drs. Slobogean and Sprague had full access to all the data in the study and take responsibility for the integrity of the data and the accuracy of the data analysis. The data that support the findings of this study are available from the corresponding author upon reasonable request.
